# From Bench to Bedside: Translating the Prolactin/Vasoinhibin Axis

**DOI:** 10.3389/fendo.2017.00342

**Published:** 2017-12-11

**Authors:** Jakob Triebel, Maria Ludivina Robles-Osorio, Renata Garcia-Franco, Gonzalo Martínez de la Escalera, Carmen Clapp, Thomas Bertsch

**Affiliations:** ^1^Institute for Clinical Chemistry, Laboratory Medicine and Transfusion Medicine, Nuremberg General Hospital, Paracelsus Medical University, Nuremberg, Germany; ^2^Facultad de Ciencias Naturales, Universidad Autónoma de Querétaro (UAQ), Querétaro, México; ^3^Instituto Mexicano de Oftalmología (IMO), I.A.P., Querétaro, México; ^4^Instituto de Neurobiología, Universidad Nacional Autónoma de México (UNAM), Querétaro, México

**Keywords:** vasoinhibins, 16K prolactin, diabetic retinopathy, diabetic macular edema, peripartum cardiomyopathy, levosulpiride, bromocriptine, dopamine D2 receptor

## Abstract

The prolactin/vasoinhibin axis defines an endocrine system, in which prolactin (PRL) and vasoinhibins regulate blood vessel growth and function, the secretion of other hormones, inflammatory and immune processes, coagulation, and behavior. The core element of the PRL/vasoinhibin axis is the generation of vasoinhibins, which consists in the proteolytic cleavage of their precursor molecule PRL. Vasoinhibins can interact with multiple different partners to mediate their effects in various tissues and anatomical compartments, indicating their pleiotropic nature. Based on accumulating knowledge about the PRL/vasoinhibin axis, two clinical trials were initiated, in which vasoinhibin levels are the target of therapeutic interventions. One trial investigates the effect of levosulpiride, a selective dopamine D2-receptor antagonist, on retinal alterations in patients with diabetic macular edema and retinopathy. The rationale of this trial is that the levosulpiride-induced hyperprolactinemia resulting in increased retinal vasoinhibins could lead to beneficiary outcomes in terms of a vasoinhibin-mediated antagonization of diabetes-induced retinal alterations. Another trial investigated the effect of bromocriptine, a dopamine D2-receptor agonist, for the treatment of peripartum cardiomyopathy. The rationale of treatment with bromocriptine is the inhibition of vasoinhibin generation by substrate depletion to prevent detrimental effects on the myocardial microvascularization. The trial demonstrated that bromocriptine treatment was associated with a high rate of left ventricular recovery and low morbidity and mortality. Therapeutic interventions into the PRL/vasoinhibin axis bear the risk of side effects in the areas of blood coagulation, blood pressure, and alterations of the mental state.

## Background

The prolactin/vasoinhibin axis defines an endocrine system, in which the pituitary secretion of prolactin (PRL), proteases at the central and peripheral level, and vasoinhibins at the target tissue level and in the circulation act in concert to regulate blood vessel growth and function, the secretion of other hormones, inflammatory and immune processes, coagulation, and behavior (1–5). The core element of the PRL/vasoinhibin axis is the generation process of vasoinhibins, which consists in the proteolytic cleavage of their precursor molecule PRL, the pituitary hormone essential for lactation and colloquially referred to as the “nursing hormone.” This cleavage, depending on the molecular site, removes a varying number of amino acid residues near the C-terminal end of uncleaved PRL, which corresponds to removal of at least the fourth alpha-helix of full-length PRL (6, 7). The remaining N-terminal residues assume a new, not yet resolved solution structure, and a new, unique array of endocrine, paracrine, and autocrine effects distinct from PRL (8). As the inhibition of angiogenesis was the first discovered effect, these molecules were named vasoinhibins (7, 9, 10). The generation, secretion, and regulation of vasoinhibin action integrates the hypothalamus, the pituitary, and the target tissue levels, which led to the description of the PRL/vasoinhibin axis that shares its overarching organizational principles with other endocrine axes (2). Vasoinhibins comprise a family of peptides, as multiple isoforms with variation in the number of amino acids and molecular mass, respectively, are present. The total number of vasoinhibins has yet to be determined, as well as their receptor binding sites, receptors, and complete signaling mechanisms, which are only partially known (1, 2, 4, 11, 12). Vasoinhibins act through a still-unidentified binding site in endothelial cell membranes which is distinct from the PRL-receptor (13) and can interact with multiple different partners to mediate their effects (2, 5, 14, 15). This interaction varies with the diverse effects in various tissues and anatomical compartments, indicating the pleiotropic nature of vasoinhibins (1, 2, 4). The regulation of blood vessels by PRL and vasoinhibins has been reviewed (1, 4, 12).

The accumulation of knowledge about the functions and effects of the PRL/vasoinhibin axis from basic studies has reached a critical mass which has triggered translation from bench to bedside and back, at present culminating in two clinical studies in which the PRL/vasoinhibin axis is target of therapeutic interventions to treat diabetic retinal diseases and peripartum cardiomyopathy (PPCM). It is the purpose of this review to discuss the principles behind these clinical studies, to address further areas of clinical relevance, to identify major barriers and clinical problems, and to point to solutions with which they could be overcome.

## Diabetic Retinopathy and Diabetic Macular Edema

**Figure 1:**
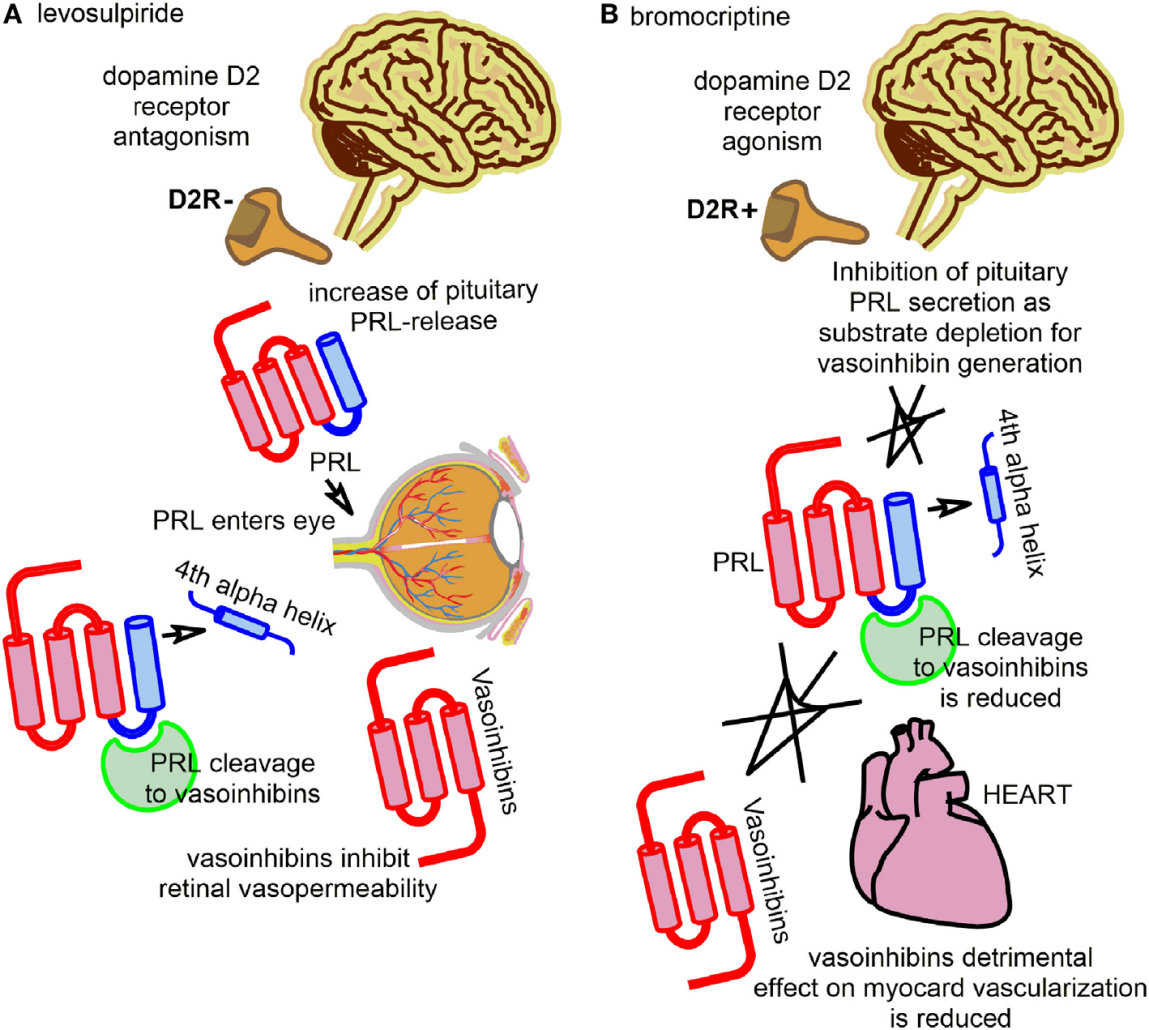
The figure illustrates the key principles employed by two current clinical studies with interventions into the regulation of the prolactin/vasoinhibin axis axis. **(A)** One trial evaluates the effect of levosulpiride on retinal outcomes in patients with diabetic macular edema and diabetic retinopathy. Levosulpiride, a dopamine D2-receptor antagonist, is used to induce an increase of pituitary prolactin (PRL)-secretion *via* antagonization of the inhibiting effect of dopamine on PRL-secretion (disinhibition of PRL-release). PRL can enter the eye and be cleaved to vasoinhibins, with beneficial effects in retinal outcomes in terms of reducing retinal vasopermeability and their vascular endothelial growth factor-antagonism. **(B)** Another trial evaluated the effect of bromocriptine on the left ventricular function in patients with peripartum cardiomyopathy. Bromocriptine was used to inhibit pituitary PRL-secretion by dopamine D2-receptor agonism. Vasoinhibins can no longer be produced by proteolytic cleavage of PRL, and their detrimental effect of the vascularization of the myocard is reduced.

The diabetogenic action of the pituitary has been described by Houssay and collaborators (16, 17). This seminal work was awarded by the Nobel Prize in Physiology or Medicine in 1947. A role of pituitary hormones in the etiopathology of retinal alterations emerged after observations of regression of diabetic retinopathy in a patient with Sheehan’s Syndrome in 1953 (18). This has led to the use of therapies against diabetic retinopathy targeting the pituitary gland by stalk section or surgical ablation, a path which, despite beneficial retinal effects, was fortunately soon abandoned (19, 20). The beneficial retinal effects after stalk section, pituitary ablation, or Sheehan’s Syndrome were, for the most part, attributed to declining levels of growth hormone and IGF-1, but circulating PRL levels were also subject of investigations addressing the etiopathology of diabetic retinopathy (21, 22). However, the results of these studies were inconsistent and did not provide sufficient mechanistic insight to delineate the actions of PRL in the diseased retina. The discovery of vasoinhibins, however, provided a new mechanistic framework and led to the reassessment of the role of PRL in the retina and its diseases. This reassessment was primarily based on the knowledge of the effects of vasoinhibins on blood vessel growth, permeability, and dilation, which correspond well with major pathological features seen in diabetic retinopathy and diabetic macular edema, for example, vascular leakage, retinal edema, intraretinal and vitreal hemorrhages, and retinal neovascularizations. How the PRL/vasoinhibin axis performs control over blood vessel growth and function at the molecular level has been the subject of two reviews, and should, therefore, not be discussed here further, but there are underlying key elements of the PRL/vasoinhibin axis at the integrative and systemic levels that are helpful for understanding ongoing clinical trials (1, 4). One of these clinical trials investigates the effect of levosulpiride on retinal alterations in patients with diabetic retinopathy and diabetic macular edema (ClinicalTrials.gov Identifier: NCT03161652). Levosulpiride, an atypical neuroleptic agent, is a benzamide derivate and a selective dopamine D2-receptor antagonist, and treatment with levosulpiride is frequently associated with the development of hyperprolactinemia. The development of hyperprolactinemia with levosulpiride is due to blockage of dopamine receptors on the pituitary lactotrophs mediating inhibition of PRL-release (23). A low dose of levosulpiride is used as a prokinetic agent (24–26). Levosulpiride-induced hyperprolactinemia is usually an unintended side effect and can be accompanied by decreased libido, erectile dysfunction in men, and galactorrhea and amenorrhea in women. The clinical study on the effect of levosulpiride on retinal alterations in patients with diabetic retinopathy and diabetic macular edema, however, is an attempt to exploit positive effects of hyperprolactinemia, induced by a low dose of levosulpiride, on retinal outcomes. The principal finding that led to the development of this concept was a study in rats, in which the induction of hyperprolactinemia resulted in vasoinhibin accumulation in the retina and a reduction of vascular endothelial growth factor (VEGF)- and diabetes-induced retinal vasopermeability was demonstrated (27). The effect could not be observed in rats with genetic deletion of the PRL-receptor; also, the effects could be blocked by bromocriptine, which lowered the levels of circulating PRL and retinal vasoinhibins. Thus, the study indicated that circulating PRL can be incorporated into the eye and cleaved to vasoinhibins intraocularly, which could lead to beneficiary outcomes in terms of a vasoinhibin-mediated antagonization of VEGF- and diabetes-induced retinal vasopermeability (Figure 1A; Table 1). In consequence, it appeared that the counteraction of angiogenic factors, such as VEGF, and of excessive vasopermeability by the raising of ocular vasoinhibins, constitute direct therapeutic interventions into pathological pathways associated with the development of diabetic retinopathy and diabetic macular edema. The development of this trial is also embedded into a long history of studies portraying the eye and its structures as targets for PRL and vasoinhibins (14, 28–40). The completion of this randomized, placebo-controlled clinical trial, which is carried out in Mexico and currently in the recruiting phase, will demonstrate whether this concept can safely and effectively be translated to its clinical application.

**Table 1:** Current clinical studies with interventions into to the regulation of the prolactin/vasoinhibin axis.

Disease	Clinical pathology	Proposed pathomechanism	Therapeutic intention	Therapeutic strategy	Drug	ClinicalTrials.gov Identifier
Diabetic retinopathy and diabetic macular edema	Retinal edema	Increase in retinal vasopermeability	Inhibition of retinal vasopermeability by vasoinhibins	Increase PRL-secretion by dopamine D2-receptor antagonism	Levosulpiride	NCT03161652

Peripartum cardiomyopathy	Low left ventricular ejection fraction	Vasoinhibin-mediated damage of myocardial vascularization	Inhibition of vasoinhibin generation in the heart	Inhibition of PRL-secretion by dopamine D2-receptor agonism	Bromocriptine	NCT00998556

## Peripartum Cardiomyopathy

A role for PRL in the etiopathology of heart failure, and PPCM in particular, was suggested in case reports in 1979 and 1984 (41, 42). However, the data remained inconclusive, particularly because a mechanism by which PRL could exert detrimental effects on the heart was not known. The discovery of cleaved PRL in 1980 in rats (43, 44) and its detection in humans 1985 (45), the identification of the anti-angiogenic effects of a 16 kDa PRL fragment in the early 1990s (9, 10), the generation of a 16 kDa PRL by cathepsin D (46), the discovery of more anti-angiogenic PRL-fragments (7, 47), and their subsequent classification as vasoinhibins (7, 48, 49), provided the framework for a study from 2007 (50), in which it was suggested that an excessive generation of vasoinhibins in the heart could impair the myocardial microvascularization and contribute to the development of PPCM. Indeed, PRL levels during the peripartum/postpartum period can be up to 20 times higher than normal, in order to facilitate lactation (51). This appears as a precondition for a vasoinhibin-related onset of PPCM, as PRL is the immediate precursor molecule of vasoinhibins, and high substrate (PRL) availability favors the enzymatic generation of vasoinhibins. A second precondition for the excessive vasoinhibin generation in PPCM appears to be a high activity of the PRL-cleaving enzyme cathepsin D, which, in combination with the elevated PRL levels, is proposed to lead to abnormally high vasoinhibin values in the heart, detrimental effects on the vascularization of the myocardium, and subsequent development of heart insufficiency. It is reported that vasoinhibins lead to an increased level of microRNA-146a expression in endothelial cells, which exerts angiostatic effects and impairs the metabolic activity of cardiomyocytes (52). More detailed molecular descriptions of the pathways, including information on possible factors involved in the myocardial signal transduction of vasoinhibins, can be found in the original papers (50, 52) and have also been reviewed (53). Based on these insights, a new therapeutic approach for PPCM was developed, using the dopamine D2-receptor agonist bromocriptine; a drug usually applied in patients with a prolactinoma or Parkinson’s disease. The principle behind this approach is the inhibition of vasoinhibin generation by substrate depletion, or the inhibition of pituitary PRL-secretion by lactotrophs, respectively (Figure 1B; Table 1). Pilot studies using bromocriptine as an add-on treatment to standard heart failure therapy reported possible beneficial effects with a normalization of left ventricular functions and dimensions (54–56). A proof-of-concept study for the evaluation of bromocriptine appeared to confirm the positive effects of bromocriptine, and a randomized, controlled multicenter clinical trial to evaluate the effect of bromocriptine in patients with PPCM, conducted in Germany, was then initiated (ClinicalTrials.gov Identifier: NCT00998556) (57, 58). The trial has recently been completed and the results demonstrated that bromocriptine treatment was associated with a high rate of left ventricular recovery and low morbidity and mortality (59).

## Potential Risks Associated with Therapeutic Interventions

Therapeutic intervention of the PRL/vasoinhibin axis is likely to be associated with risks that complicate the clinical decision to commence therapy with both D2R-antagonists and agonists, or stimulating/blocking vasoinhibin generation and/or signaling by other means (60). These risks can be inferred from the known profile of biological effects of vasoinhibins, but may also include unexpected side effects and complications that can only be identified in clinical studies. Relevant issues, for example, are the effects of vasoinhibin stimulation or blockage on blood coagulation, as well as possible effects on blood pressure. Plasminogen activator inhibitor-1 was recently identified as a frequent binding partner of vasoinhibins, and this binding is responsible for the mediation of profibrinolytic effects of vasoinhibins (5). Blocking vasoinhibin production and/or signaling could, therefore, contribute to the formation or stabilization of thrombi. Of note, histological analysis of lung sections demonstrated a higher number of thrombi in control mice than in vasoinhibin treated mice (5). The clinical relevance of this observation is—at present—unclear, but it points to the importance of vigilance toward thrombotic events in patients in which inhibition of vasoinhibin generation and/or signaling is the target of intervention, for example, when inhibiting vasoinhibin generation with bromocriptine in patients with PPCM (60). Likewise, elevating PRL and/or vasoinhibin levels, as in the trial evaluating levosulpiride for the treatment of diabetic macular edema and retinopathy, could include delayed and disturbed coagulation. In mice, vasoinhibins have been demonstrated to be able to upregulate blood pressure by modulating the activity of endothelial nitric oxide synthase (eNOS) (61). Hence, blood pressure fluctuations may be due to changes in vasoinhibin levels and could appear when vasoinhibin levels are manipulated. Indeed, some of the cardiovascular side effects of bromocriptine, such as hypotension, syncope, and pleural/pericardial effusion, could be influenced by a decline of vasoinhibin levels (60).

The range of possible side effects when intervening the PRL/vasoinhibin axis also includes effects on the mental state. These effects are implied by experiments in rodents, demonstrating that the intraventricular administration of vasoinhibins leads to an increase in anxiety and depression-related behaviors (3). This scenario is further implied by an investigation showing a high prevalence of depression in women with PPCM, as the higher circulating vasoinhibin levels in these patients may enter the cerebrospinal fluid and exert neuropeptide-like effects in the central nervous system (62, 63). Lastly, the occurrence of maniac episodes after the initiation of medication with cabergoline and bromocriptine (64) may be related to central vasoinhibin levels, as a sudden decline of vasoinhibins may contribute to elevated arousal and affect (63). Of note, Ergot-derived drugs, such as bromocriptine, can induce retroperitoneal fibrosis and pleural, pericardial, and cardiac valve fibrotic reactions (65).

## Major Barriers

The major barrier not yet overcome, which delays a more thorough, more in-depth clinical evaluation of vasoinhibins is the lack of a quantitative assay for the determination of vasoinhibins in biological fluids, such as serum, plasma, cerebrospinal fluid, urine, and tissue homogenates. Some experimental techniques, such as mass spectrometry, have been evaluated, but the only more widely used methodology for detecting vasoinhibins is immunoprecipitation with anti-PRL antibodies and subsequent Western blotting (28, 50, 66). This technique has multiple limitations, including a relatively low sensitivity and a relative lack of quantitative information, and is, in most cases, not precise enough to unambiguously discriminate between vasoinhibin isoforms. The presence of multiple vasoinhibin isoforms of different molecular masses is a challenge for the development of a quantitative immunoassay, as it complicates the decision of which isoform should be targeted when monoclonal anti-vasoinhibin antibodies are manufactured. This challenge would be alleviated, if there would only be one dominating isoform being associated with a particular disease, such as PPCM or preeclampsia, and the other isoforms would not be produced, or only be present in negligible amounts. However, in contrast to *in vitro* and *in vivo* experimental studies, no clinical study has provided clear proof of the exact identity of the vasoinhibin isoform under investigation, that is their complete amino acid sequence or cleavage site within the PRL sequence, which could then be used as the template to produce monoclonal anti-vasoinhibin antibodies. Moreover, several clinical studies reported the association of changes in vasoinhibin levels of more than one isoform at the same time, indicating that, according to disease state, more than one isoform may be involved (28, 67). These observations extend to another unmet challenge requiring attention: the site of vasoinhibin production and the controlling mechanisms determining their overall isoform composition. Vasoinhibins are generated in the pituitary gland and in multiple peripheral tissues and fluids (2, 68), but information about which of these sites is the one producing vasoinhibins measured in the circulation of patients is not available. For example, elevated serum levels of vasoinhibins in patients with PPCM might derive from PRL cleavage occurring in the heart, but may also originate from another site of vasoinhibin generation. This problem is relevant for clinical investigations, as some reports correlate the serum activity of PRL-cleaving, vasoinhibin generating enzymes with circulating vasoinhibin levels, implying that vasoinhibins are either produced in the circulation, or that the enzyme activity in the circulation corresponds with its activity at the site of vasoinhibin generation, for example, at the organ or tissue level (50, 69). Both possibilities are not supported by evidence and, thus, require clarification. Moreover, questions about the controlling mechanisms of single vasoinhibin isoforms production arise when only one cathepsin D-, or MMP-cleaved isoform, is detected (50). These enzymes use multiple cleavage sites within the PRL sequence to generate vasoinhibins of varying molecular mass, and if only a single isoform is produced, unknown controlling mechanisms must be in place suppressing the generation of the other isoforms (70). Of note, the quantitative determination of vasoinhibin levels is a missing piece in the characterization of the role of vasoinhibins in diabetic retinopathy and PPCM, but also in other diseases that have been brought into context with a dysregulation of vasoinhibins, and only if vasoinhibin levels are evaluated, their role in the aforementioned diseases can be further substantiated.

## Perspectives

The present time is unique in the scientific history of PRL research, as new entities—diabetic retinal diseases and PPCM—are added to the short list of conditions in which the pituitary secretion of PRL is target of therapeutic interventions. This list had previously comprised only the condition of prolactinoma and the inhibition or PRL-release for ablactation or secondary amenorrhea. Of note, there are more clinical entities in which studies reported that a dysregulation of PRL and of the PRL/vasoinhibin axis might play a role, for example, breast and prostate cancer (71–75), preeclampsia and eclampsia (67, 76, 77), pregnancy-induced hypertension (78), pulmonary artery hypertension (79), retinopathy of prematurity (28), and rheumatoid arthritis (80). These conditions require thorough clinical investigation, including determination of PRL and vasoinhibin levels, and, ideally, additional experimental validation. In due course, in case the role of the PRL/vasoinhibin axis in these diseases is consolidated, it is possible that altering PRL and vasoinhibin levels represents a new option for therapeutic intervention. However, a better understanding of the physiological regulation of this axis and of its alterations under such diseases is required, as too many factors are still unclear. These factors comprise, as discussed, the sites and regulatory mechanisms involved in vasoinhibin generation, the relative contribution of vasoinhibins isoforms generated not only by proteolytic cleavage of PRL but also by the cleavage of related hormones, such as growth hormone and placental lactogen (47, 81). Undoubtedly, new information about the solution structure of vasoinhibins, their bioactive domains, receptors and signaling mechanisms, and the evolutionary emergence of the various isoforms (2, 8, 11) are required to advance the field in the future and to substantiate the impact of the PRL/vasoinhibin axis in human health and disease.

## Author Contributions

JT wrote the manuscript. MR-O, RG-F, GE, CC, and TB edited and revised the manuscript. All authors approved the final version of the manuscript.

## Conflict of Interest Statement

The authors declare that the research was conducted in the absence of any commercial or financial relationships that could be construed as a potential conflict of interest.
